# Oral cavity as a reservoir of respiratory pathogens: microbial dynamics in patients receiving mechanical ventilation

**DOI:** 10.31744/einstein_journal/2026AO2476

**Published:** 2026-08-21

**Authors:** Gabrielle Luiza de Camargos Pessoa, Ana Maria Schroden Rodrigues da Cunha, Leticia Schroden Rodrigues da Cunha, Giovanna Schroden Rodrigues da Cunha, Vinicius Rangel Geraldo-Martins, Ruchele Dias Nogueira

**Affiliations:** 1 Universidade de Uberaba Posgraduate Program in Integrated Dental Clinic Uberaba MG Brazil Posgraduate Program in Integrated Dental Clinic, Universidade de Uberaba, Uberaba, MG, Brazil; 2 Universidade de Uberaba Department of Dentistry Uberaba MG Brazil Department of Dentistry, Universidade de Uberaba, Uberaba, MG, Brazil; 3 Universidade de Uberaba Medical School Uberaba MG Brazil Medical School, Universidade de Uberaba, Uberaba, MG, Brazil

**Keywords:** Saliva, Tracheal, Body fluids, Respiration, artificial, Respiratory pathogens, Intensive care unit

## Abstract

**Objective:**

To investigate the prevalence, co-occurrence, and temporal dynamics of *Staphylococcus aureus, Klebsiella pneumoniae, Streptococcus pneumoniae, and Pseudomonas aeruginosa* in oral and tracheal samples from critically ill patients receiving mechanical ventilation.

**Methods:**

This observational analytical study included 21 adult patients receiving mechanical ventilation who were admitted to an intensive care unit. Oral and tracheal samples were collected within the first 24 h after intubation (Day 0) and again after 48 h (Day 2). Bacterial genomic deoxyribonucleic acid was extracted and analyzed using quantitative realtime polymerase chain reaction. Microbial detection was evaluated qualitatively based on the presence or absence of each microorganism and quantitatively using relative bacterial load analysis. Statistical analyses assessed correlations between oral and tracheal colonization patterns and temporal changes in bacterial load.

**Results:**

More than 43% of oral and tracheal samples tested positive for at least one investigated pathogen at both collection time points, indicating persistent microbial colonization during mechanical ventilation. Significant positive correlations for *Staphylococcus aureus* detection were observed between oral and tracheal samples on Day 0 (p=0.0028) and Day 2 (p=0.0021), suggesting a possible association between oral colonization and lower airway microbial presence. *Streptococcus pneumoniae* detected in initial oral samples showed a significant correlation with tracheal detection after 48 h (p=0.03). Quantitative analyses demonstrated no significant changes in bacterial load over time for most microorganisms. However, *Klebsiella pneumoniae* showed a significant increase in tracheal bacterial load between Day 0 and Day 2 (p=0.04), with higher tracheal loads than oral samples on Day 2 (p=0.01).

**Conclusion:**

The oral cavity may serve as an important reservoir of respiratory pathogens in patients receiving mechanical ventilation. The observed correlations between oral and tracheal colonization and pathogen-specific colonization patterns reinforce the relevance of oral health management in critical care settings. In particular, the increase in tracheal *Klebsiella pneumoniae* load suggests that lower airway proliferation may occur during mechanical ventilation independently of simultaneous increases in oral bacterial load.

## Highlights

■ Oral and tracheal pathogens persisted throughout mechanical ventilation.

■ *S. aureus* showed consistent oral-tracheal correlations at both time points.

■ Tracheal K. *pneumoniae* load increased after 48 hours and exceeded oral load.

■ The oral microbiota may contribute to lower respiratory tract colonization and systemic infection

## In Brief

This study investigated the prevalence, co-occurrence, and temporal dynamics of major respiratory pathogens in paired oral and tracheal samples from mechanically ventilated intensive care unit patients. Persistent pathogen detection and significant oral-tracheal correlations, particularly for *Staphylococcus aureus* and *Streptococcus pneumoniae,* highlight the oral cavity as a potential reservoir for lower respiratory tract and systemic infections.

## Graphical abstract

**Figure:**
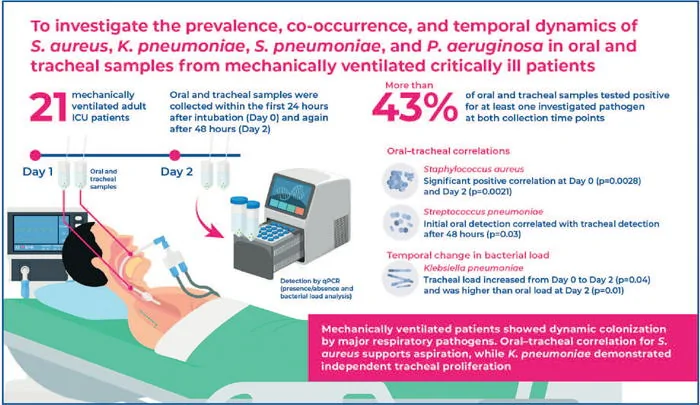

## INTRODUCTION

Patients admitted to intensive care units (ICUs) frequently require mechanical ventilation to support their respiratory function. Although this intervention is lifesaving, a significant risk of complications, most notably ventilator-associated pneumonia (VAP), remains. The prevalence of VAP is high in this patient population, making this group particularly relevant for investigation. Ventilator-associated pneumonia is a serious hospital-acquired infection of the lung parenchyma that develops in patients receiving mechanical ventilation. Ventilator-associated pneumonia is associated with increased patient morbidity, prolonged ICU stays, higher healthcare costs, and elevated mortality rates among critically ill patients.(1) The primary mechanism underlying VAP involves the aspiration of pathogenic microorganisms from the oropharynx into the lower respiratory tract.(2,3) This mechanism highlights the oral cavity as a significant reservoir for potential respiratory pathogens.

The oral microbiome in healthy individuals is a diverse and balanced ecosystem. However, the oral microbiome in critically ill patients receiving intubation is frequently disrupted, leading to dysbiosis.(4,5) Factors such as underlying systemic diseases, reduced salivary flow, impaired immune responses, and the presence of endotracheal tubes can profoundly alter the oral environment. These changes favor the proliferation of opportunistic pathogens, shifting the oral microbiota towards one dominated by potentially virulent microorganisms.(6,7,8) Periodontal disease, a chronic inflammatory condition, is also recognized as a potential source of respiratory pathogens, further emphasizing the link between oral health and systemic infections, particularly among vulnerable populations.(9,10)

Among the bacterial species most frequently implicated in VAP are *Staphylococcus aureus, Klebsiella pneumoniae, Streptococcus pneumoniae,* and *Pseudomonas aeruginosa. S. aureus* is a versatile pathogen, and its antibiotic resistance, particularly methicillin-resistant *S. aureus* (MRSA), poses a significant clinical challenge.(11) *K. pneumoniae* is known for its rapidly evolving antibiotic resistance mechanisms and its association with severe healthcare-associated infections.(12) *S. pneumoniae* is a common etiological agent of pneumonia, whereas *P. aeruginosa* is an opportunistic pathogen frequently encountered in hospital settings, known for its intrinsic resistance to many antibiotics and its ability to form biofilms.(13) The persistent presence of these bacteria, even in clinically stable patients receiving mechanical ventilation, signifies ongoing colonization and a predisposition to severe respiratory infections.

The importance of oral healthcare in preventing VAP has been widely acknowledged. Interventions such as regular tooth brushing and the use of antiseptic mouthwashes, including chlorhexidine, have been explored for their potential to reduce the bacterial load in the oral cavity and subsequently decrease the incidence of VAP.(14,15) Studies suggest that oral hygiene can reduce bacterial transport and colonization during intubation.(16,17,18,19) However, the optimal methods and consistent effectiveness of oral care in this patient population remain subjects of ongoing research and debate.(20) The unique challenges associated with delivering comprehensive oral care to patients receiving mechanical ventilation, coupled with potential adverse effects of certain agents and limitations in reaching all colonized sites,(21) necessitate a deeper understanding of bacterial dynamics in this critical population. Therefore, understanding the relationship between oral and tracheal bacterial populations is crucial for effective VAP prevention and management.

Despite increasing evidence linking oral dysbiosis to VAP, limited data are available regarding the simultaneous temporal dynamics of oral and tracheal colonization by specific respiratory pathogens in critically ill patients receiving mechanical ventilation. Understanding these microbial interactions may contribute to the development of more targeted preventive strategies.

## OBJECTIVE

To investigate the prevalence, co-occurrence, and temporal dynamics of *S. aureus, K. pneumoniae, S. pneumoniae, and P. aeruginosa* in oral and tracheal samples from critically ill patients receiving mechanical ventilation.

## METHODS

### Study design and patient enrollment

This observational analytical study adhered to ethical guidelines and received approval from the Research Ethics Committee of the *Universidade de Uberaba* -UNIUBE (CAAE: 76451423.0.0000.5145; #7.511.664). Informed consent was obtained from all participating patients or their legally authorized representatives before study enrollment. Patient recruitment was conducted in the ICU of *Hospital Regional de Uberaba,* Brazil. Inclusion criteria comprised adult patients admitted to the ICU who required orotracheal intubation and presented with detectable tracheal secretions. Exclusion criteria included a documented history of gastric content aspiration, previous thoracic surgery, pre-existing chronic obstructive pulmonary disease or other significant pulmonary comorbidities, or a confirmed diagnosis of pulmonary infection at the time of ICU admission.

Initially, samples were collected from 49 patients upon their indication for intubation. However, 28 patients were subsequently excluded from the prospective 48-h follow-up analysis. Reasons for exclusion included extubation within 24 h after intubation, death before the second sample collection, clinical instability that rendered the second collection unsafe or impractical, or withdrawal of consent by the patient or the patient's legally authorized representative. Ultimately, 21 patients (11 men and 10 women) completed the study, with a mean age of 70.7 ± 12.5 years. Patients receiving mechanical ventilation represented the target population because of their unique risk factors for VAP, making inclusion essential to achieve the study objective. At the time of both initial and follow-up sample collections, none of the included patients exhibited clinical signs or symptoms indicative of pneumonia or any other active infectious process.

### Sample collection

Oral and tracheal samples were collected at two distinct time points: Collection 1 (Day 0): Samples were obtained within the first 24 h after orotracheal intubation and included an oral swab (S1) and a tracheal swab (T1); Collection 2 (Day 2): A second set of samples, comprising an oral swab (S2) and a tracheal swab (T2), was collected approximately 48 h after intubation.

Oral sample collection (S1, S2): For oral sampling, a sterile cotton swab was gently and thoroughly rubbed across multiple oral mucosal surfaces, including the buccal mucosa, dorsal surface of the tongue, and gingival surfaces. The swab was then immersed in the patient's saliva for approximately 1 min to ensure adequate saturation with oral fluids and detached microorganisms.

Tracheal sample collection (T1, T2): Tracheal samples were acquired using a sterile cotton swab immediately after a routine tracheal aspiration procedure. The swab was carefully rubbed against the distal tip of the suction catheter. This standardized approach was designed to capture microorganisms present in tracheal secretions without directly entering the lower respiratory tract; thereby, minimizing patient discomfort and potential complications.

Immediately after collection, all swabs were immersed in 1mL of sterile phosphate-buffered saline (PBS). Each sample tube was labeled with the patient's initials, the precise date of collection, and the specific sample type (oral or tracheal). Samples were then promptly transported to the Uniube microbiology laboratory and stored at -70°C until deoxyribonucleic acid (DNA) extraction.

Following the initial sample collection, the dedicated oral health team conducted a comprehensive intraoral clinical examination for each patient. Subsequently, standardized oral hygiene protocols were rigorously implemented. These protocols included the diligent use of an oral antiseptic solution, efficient removal of secretions using a suction catheter, and topical application of 5% dexpanthenol cream to protect the patient's lips. All sample collection procedures and oral hygiene interventions were standardized through comprehensive training of the oral health team, hospital physiotherapists, and the attending dentist researchers, ensuring consistency and reliability throughout the study.

### DNA extraction

Bacterial genomic DNA was extracted from both oral and tracheal samples utilizing the PureLink Genomic DNA Mini Kit (Invitrogen™) according to the manufacturer's instructions. Briefly, 200 μL of each sample (from the PBS suspension) was carefully transferred to a sterile 1.5 mL Eppendorf tube. Subsequently, 20 μL of Proteinase K and 500 μL of PureLink Genomic Lysis/ Binding Buffer were added. The mixture was vortexed thoroughly to ensure complete homogenization and incubated at 55°C for 10 min to facilitate cell lysis.

Following incubation, the tubes were briefly centrifuged to collect any condensation from the tube caps. Subsequently, 200 μL of 100% ethanol was added to the lysate, and the mixture was vortexed for 5 s to achieve homogeneity. The entire volume (approximately 640μL) was then transferred to a PureLink Spin Column supplied with the kit. The column was centrifuged at 10,000 × g for 1 min at room temperature, and the flow-through was discarded. The spin column was then transferred to a new sterile collection tube.

For DNA washing, 500μL of Wash Buffer 1 (prepared with 100% ethanol according to the manufacturer's instructions) was added to the column, followed by centrifugation at 10,000 × g for 1 min at room temperature. The flow-through was discarded. Subsequently, 500 μL of Wash Buffer 2 was added, and the column was centrifuged at maximum speed for 3 min at room temperature to thoroughly dry the membrane. The flow-through and collection tube were discarded.

Finally, for DNA elution, the column was transferred to a clean sterile 1.5mL Eppendorf tube. A volume of 100μL of PureLink Genomic Elution Buffer was added directly to the center of the column membrane, followed by centrifugation at maximum speed for 1 min at room temperature. The filter column was then discarded, and the purified DNA solution was promptly stored at -20°C until quantitative real-time polymerase chain reaction (qPCR) analysis.

### Quantitative real-time polymerase chain reaction

qPCR was performed using the QuantiNova SYBR Green PCR Kit (Qiagen) on a StepOne Real-Time PCR System. Oligonucleotide primers (Table 1) were reconstituted in 1 × TE buffer (l0 mM Tris-HCl and 1 mM ethylenediaminetetraacetic acid [EDTA], pH 7.5-8.0) to the appropriate working concentrations. Each 20 μL reaction mixture comprised 4 μL of extracted DNA, 10 μL of SYBR Green Master Mix, 1 μL of forward primer (F), 1 μL of reverse primer (R), and 4 μL of ultrapure water. Reactions were prepared in 96-well plates. The thermocycling conditions consisted of an initial denaturation step at 95°C for 3 min, followed by 40 cycles of denaturation at 95°C for 30 s, primer annealing at 58°C for 30 s, and extension at 72°C for 30 s. To confirm primer specificity and the absence of nonspecific amplification products, a dissociation curve analysis was routinely performed immediately after the amplification cycles. Real-time detection of fluorescence signals allowed for the quantitative assessment of bacterial genetic material, which was then converted into a relative bacterial load (expressed as cell number equivalents) using a standard curve previously prepared with known concentrations of bacterial genomic DNA.

**Table 1:** Oligonucleotide primers used for quantitative real-time polymerase chain reaction

Target	Primer sequence (5'-3')
Universal 16S rRNA	
F	TGGAGCATGTGGTTTAATTCGA
R	TGCGGGACTTAACCCAACA
S. aureus	
F	AACAGTATATAGTGCAACTTCAA
R	CTTTGTCAAACTCGACTTCAA
S. pneumoniae	
F	CACTCAACTGGGAATCCGC
R	CCAGGCACCATTATCAACAGG
P aeruginosa	
F	GTTCGGCCTGTTGCCTAAG
R	AACTCGTGCTGCTTTCGC
K. pneumoniae	
F	TACACAATCGCCCGTTGAAC
R	CCCGGTTAGATCCATGGTGA

### Statistical analysis

qPCR results were analyzed both qualitatively (presence or absence) and quantitatively using relative bacterial load values derived from fluorescence amplification curves. Cycle threshold (Ct) values were inversely proportional to bacterial load and were converted into relative cell number equivalents using standard calibration curves. Statistical analyses were performed using BioEstat software version 5.3 (Optical Digital Technology, Belém, PE, Brazil). Initial descriptive statistics were generated to characterize the study population and summarize the absolute and relative frequencies of bacterial detection. Associations between the presence or absence of each bacterial species across collection sites (oral *versus* tracheal) and collection time points (Day 0 *versus* Day 2) were evaluated using Fisher's exact test or the χ^2^ test, as appropriate. Pearson correlation analysis was used to assess correlations between bacterial detection or relative bacterial loads across collection sites and time points. Differences in relative bacterial loads were evaluated using the appropriate analysis of variance test. A two-sided p<0.05 was considered statistically significant.

## RESULTS

Of the 49 patients initially screened for intubation in the ICU of *Hospital Regional de Uberaba* (25 women and 24 men), 21 patients (11 men and 10 women) with a mean age of 70.7 years (standard deviation [SD]: 12.5) met the criteria for prospective analysis. The remaining 28 patients were excluded because of early extubation (within 24 h after intubation), death, clinical instability on the day of follow-up sample collection, or withdrawal of consent. Importantly, none of the 21 patients receiving mechanical ventilation who were included in the study exhibited clinical signs or symptoms of pneumonia or any other active infectious process at either the initial (S1, T1) or 48-h (S2, T2) collection time points.

Oral and tracheal samples from these 21 patients were analyzed using PCR to detect *S. aureus, K. pneumoniae, S. pneumoniae,* and *P. aeruginosa,* all of which are frequently implicated in VAP. Table 2 summarizes the detection frequencies of these microorganisms in the collected samples. Statistical analysis revealed no significant differences in the overall detection (presence or absence) of these bacteria when comparing collection time points or sample types (p>0.09, q<1.56). These findings indicate persistent detection of these pathogens in both oral and tracheal samples throughout the study period.

**Table 2:** Detection frequencies of bacterial species in oral (S) and tracheal (T) samples at time points 1 and 2 (n=21)

Bacteria	Detection	S1	S2	T1	T2
		n (%)	n (%)	n (%)	n (%)
*S. aureus*	Yes	10(48)	9 (43)	10(48)	11 (52)
	No	11 (52)	12 (57)	11 (52)	10(48)
*K. pneumoniae*	Yes	13 (62)	10(48)	10(48)	12(57)
	No	8 (38)	11 (52)	11 (52)	9 (43)
*S. pneumoniae*	Yes	14 (67)	13 (62)	10(48)	10(48)
	No	7 (33)	8 (38)	11 (52)	11 (52)
*P. aeruginosa*	Yes	11 (52)	14 (67)	11 (52)	10(48)
	No	10(48)	7 (33)	10(48)	11(52)

Despite the absence of clinical infection, more than 43% of the analyzed oral and tracheal samples tested positive for at least one of the target bacteria at both collection time points. Notably, most initial oral samples were positive. Although *P. aeruginosa* showed increased detection in oral samples from the first to the second collection, the remaining bacterial species generally exhibited decreased oral detection over the 48-h period.

Figure 1 illustrates the positive (gray) and negative (white) detection of bacteria in oral (S1/S2) and tracheal (T1/T2) samples from the 21 patients at both collection time points. Correlation analysis of the presence or absence of *S. aureus* between oral and tracheal samples revealed a significant positive correlation at both the initial collection (S1 *versus* T1; p=0.0028, r=0.61) and the 48-h collection (S2 *versus* T2; p=0.0021, r=0.63). Specifically, *S. aureus* was detected in both S1 and T1 samples from eight patients, whereas another eight patients showed no detection in either. Furthermore, a positive correlation for *S. aureus* was also observed between the initial and 48-h oral samples (S1 *versus* S2; p=0.01, r=0.52), with seven patients remaining positive and eight remaining negative at both time points. This longitudinal correlation was not observed between the initial and 48-h tracheal samples (p=0.59, r=0.14).

**Figure 1:**
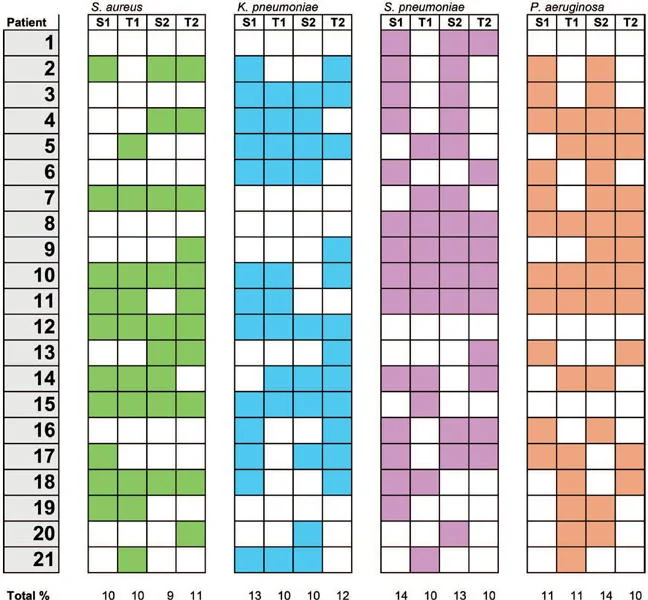
Positive (gray) and negative (white) bacterial detection in oral (S) and tracheal (T) samples at time points 1 and 2 (n=21)

For *K. pneumoniae,* a significant positive correlation in detection was found only at the initial collection between oral and tracheal samples (S1 *versus* T1; p=0.009, r=0.55), with nine patients showing detection in both samples and seven showing no detection in either sample. No other significant correlations for *K. pneumoniae* were observed among S1, S2, T1, and T2 (p>0.11, r>0.05).

In contrast, no significant positive correlations were observed for the detection of *P. aeruginosa* and *S. pneumoniae* across most oral and tracheal samples or collection time points (S1, S2, T1, and T2; p>0.11, r>0.05). However, a significant positive correlation was identified for *S. pneumoniae* when comparing detection in the initial oral samples (S1) with detection in the 48-h tracheal samples (T2; p=0.03, r=0.47). Specifically, six patients showed no detection in either S1 or T2, whereas nine showed detection in both samples. No similar delayed correlation was observed for *S. aureus* (p=0.13, r=0.33), *K. pneumoniae* (p=0.16, r=0.31), or *P. aeruginosa* (p=0.13, r=0.33).

Figure 2 illustrates the mean fluorescence intensity, representing the relative bacterial load, for each detected bacterium in oral and tracheal samples. Quantitative analysis of relative bacterial load revealed no statistically significant differences for *S. aureus* when comparing S1 and S2 (p=0.10), T1 and T2 (p=0.63), S1 and T1 (p=0.16), S2 and T2 (p=0.59), or S1 and T2 (p=0.19). Similarly, *S. pneumoniae* showed no significant differences in relative bacterial load between S1 and S2 (p=0.42), T1 and T2 (p=0.70), S1 and T1 (p=0.12), S2 and T2 (p=0.06), or S1 and T2 (p=0.14). *P aeruginosa* also demonstrated no significant differences across these comparisons (p>0.36). In contrast, *K. pneumoniae* showed a statistically significant increase in relative bacterial load in tracheal samples between T1 and T2 (p=0.04). Furthermore, the relative bacterial load of *K. pneumoniae* was significantly higher in T2 samples than in S2 samples (p=0.01). No significant differences were found for *K. pneumoniae* between S1 and S2 (p=0.26), S1 and T1 (p=0.27), or S1 and T2 (p=0.72). These findings indicate a specific increase in *K. pneumoniae* relative bacterial load within tracheal samples during the 48-h study period.

**Figure 2:**
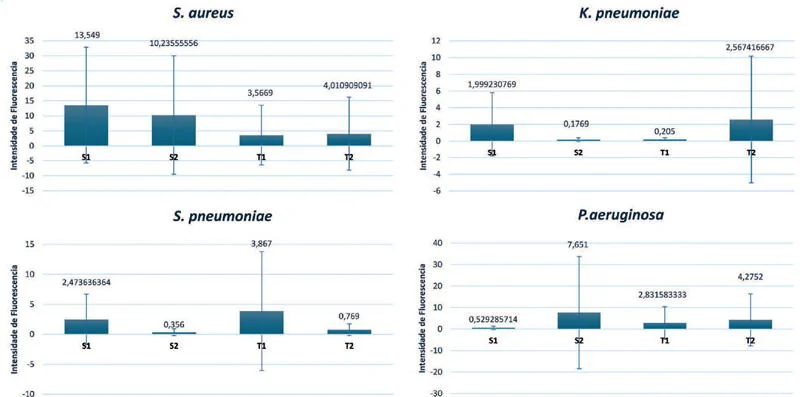
Mean fluorescence intensity of detected bacterial species in oral and tracheal samples

## DISCUSSION

This study investigated the presence, detection patterns, and relative bacterial load of key respiratory pathogens—*S. aureus, K. pneumoniae, S. pneumoniae,* and *P. aeruginosa*—in oral and tracheal samples from clinically stable patients receiving mechanical ventilation. The study population is particularly relevant because mechanical ventilation creates specific risk factors for VAP. A significant finding was that more than 43% of patients tested positive for at least one target bacterium at both the initial and 48-h sample collections despite the absence of clinical signs of infection. This high prevalence is consistent with previous research identifying the oral cavity as an important reservoir of opportunistic pathogens in critically ill hospitalized patients.(7,8) Such colonization represents a continuous risk for the development of VAP.(1) Initially, oral samples (S1) showed high detection rates for *K. pneumoniae* (62%), *S. pneumoniae* (67%), and *S. aureus* (48%). Although the detection rate of *P. aeruginosa* increased in oral samples from S1 to S2 (52% to 67%), the remaining bacterial species generally showed decreased oral detection during the 48-hour study period.

Despite these individual changes, statistical analysis revealed no significant differences in the overall detection (presence or absence) of these bacteria across collection time points or sample types (p>0.09, q<1.56). These findings indicate persistent colonization, suggesting that although the detection of individual bacterial species may fluctuate, both oral and tracheal sites remain colonized. The oral microbiota of patients receiving mechanical ventilation may undergo dysbiosis,(4,5) and the presence of these microorganisms within the oral biofilm has been well documented.(7,2) Correlation analyses evaluating bacterial presence or absence provided additional insights into the potential relationships between oral and tracheal colonization.

A significant positive correlation for *S. aureus* was observed between oral and tracheal samples at both the initial (S1 *versus* T1; p=0.0028, r=0.61) and 48-h (S2 *versus* T2; p=0.0021, r=0.63) collections. This association, with *S. aureus* detected in both oral and tracheal samples from eight patients at S1/T1 and absent from both samples in another eight patients, strongly supports the hypothesis that aspiration may contribute to the transfer of pathogens to the lower respiratory tract.(3,2) Furthermore, a positive correlation for *S. aureus* detection was found between the initial and 48-h oral samples (S1 *versus* S2; p=0.01, r=0.52), indicating relatively stable oral colonization over time. However, a similar association was not observed between the initial and 48-h tracheal samples (T1 *versus* T2; p=0.59, r=0.14), suggesting that tracheal colonization may be influenced by factors beyond persistent oral colonization.

For *K. pneumoniae,* a significant positive correlation was observed only between oral and tracheal samples collected at the initial time point (S1 *versus* T1; p=0.009, r=0.55). The absence of significant correlations across the remaining sample comparisons (p>0.11, r>0.05) suggests that the relationship between oral and tracheal colonization by *K. pneumoniae* may be more complex after the initial colonization. Conversely, *P. aeruginosa* and *S. pneumoniae* generally showed no significant positive correlations between oral and tracheal detection or across collection time points (p>0.11, r>0.05). However, a significant positive correlation was identified between *S. pneumoniae* detection in the initial oral samples (S1) and 48-h tracheal samples (T2; p=0.03, r=0.47). This delayed association suggests that oral colonization by *S. pneumoniae* may contribute to subsequent tracheal colonization in patients receiving mechanical ventilation.(9)

Oral biofilm can serve as a reservoir for these microorganisms, which may subsequently be aspirated into the lower respiratory tract.(7,2) Beyond the presence or absence of bacterial detection, quantitative analysis of relative bacterial load revealed distinct patterns. For *S. aureus, S. pneumoniae,* and *P. aeruginosa,* no statistically significant differences in relative bacterial load were observed across the evaluated comparisons (p>0.06). These findings suggest that the relative bacterial load of these pathogens remained stable during the 48-h study period in clinically stable patients. However, a significant finding emerged for *K. pneumoniae.* The relative bacterial load was significantly higher in 48-h tracheal samples (T2) than in both the initial tracheal samples (T1; p=0.04) and 48-h oral samples (S2; p=0.01). These findings suggest an increase in *K. pneumoniae* bacterial load within tracheal samples during mechanical ventilation, despite the absence of a corresponding increase in oral samples.

The identification of persistent patterns of oral and tracheal colonization reinforces the importance of integrating oral healthcare protocols into ICU management. Moreover, the observed increase in tracheal *K. pneumoniae* relative bacterial load suggests that standard oral hygiene measures alone may not fully prevent lower airway colonization in patients receiving mechanical ventilation. Although maintaining good oral hygiene is fundamental for reducing bacterial load and preventing VAP,(6,14,15,16,17,18,19) the present findings indicate that *K. pneumoniae* may exhibit distinct colonization dynamics within the lower respiratory tract.(12,13) This dynamic increase in tracheal relative bacterial load may justify closer microbiological surveillance and future investigation of targeted preventive strategies for *K. pneumoniae*-associated VAP, particularly considering the inconsistent effectiveness reported for some oral care interventions, including chlorhexidine mouthwash.(20) The potential use of topical antibiotics within the oral cavity to reduce bacterial migration into the lower respiratory tract also remains an area of ongoing investigation.(21)

This study has several limitations that should be acknowledged, including the relatively small sample size, the single-center design, and the short 48-h follow-up period. Additionally, molecular detection by qPCR does not distinguish viable from non-viable bacteria and does not allow assessment of antimicrobial resistance profiles. Therefore, larger longitudinal studies integrating microbiological culture and sequencing approaches are warranted.

## CONCLUSION

In summary, this study demonstrated a high prevalence and dynamic colonization patterns of *S. aureus, K. pneumoniae, S. pneumoniae,* and *P. aeruginosa* in patients receiving mechanical ventilation, even in the absence of clinical signs of infection. Significant correlations between oral and tracheal detection of *S. aureus* support a potential association between oral colonization and lower airway microbial presence. Quantitative analysis further demonstrated a significant increase in the relative bacterial load of *K. pneumoniae* in tracheal samples during the study period, suggesting distinct colonization dynamics for this pathogen. These findings reinforce the relevance of oral health management in patients receiving mechanical ventilation and suggest that pathogen-specific microbial behavior should be considered when developing future preventive strategies for ventilator-associated pneumonia. Future strategies must integrate these insights to develop precise risk stratification tools and targeted prophylactic measures, ultimately enhancing patient safety and outcomes in critical care.

## Data Availability

After publication, data will be available from the authors upon request - this condition is justified in the manuscript. Due to ethical restrictions involving patient data, the dataset is not publicly available but may be obtained from the authors upon reasonable request.
